# Factors influencing infant feeding for Aboriginal and Torres Strait Islander women and their families: a systematic review of qualitative evidence

**DOI:** 10.1186/s12889-022-14709-1

**Published:** 2023-02-09

**Authors:** Fiona Mitchell, Troy Walker, Karen Hill, Jennifer Browne

**Affiliations:** 1 Deakin Rural Health, School of Medicine, Deakin University, PO Box 423, 3280 Warrnambool, VIC Australia; 2 Institute of Physical Activity and Nutrition, School of Exercise and Nutrition Sciences, Deakin University, 221 Burwood Highway, 3125 Burwood, VIC Australia; 3 Institute for Health Transformation, School of Health and Social Development, Deakin University, Locked Bag 20000, 3220 Geelong, VIC Australia

**Keywords:** Aboriginal and Torres Strait Islander, Indigenous, Infant feeding, Breastfeeding, Nutrition, Ecological model, Qualitative research

## Abstract

**Background:**

Breastfeeding provides all the necessary energy and nutrients for an infant and provides many benefits for mothers and babies. The effects of colonisation have contributed to reduced prevalence and duration of breastfeeding among Australian Aboriginal women and widespread use of infant formula as a substitute for breastmilk. This review aimed to synthesise qualitative evidence about the factors that influence breastfeeding and infant feeding practices of Aboriginal and Torres Strait Islander women and their families.

**Methods:**

MEDLINE, CINAHL, Informit and Google Scholar were systematically searched for qualitative studies that included the perspective of Aboriginal and Torres Strait Islander women and their families about the factors influencing infant feeding decisions. Included studies were appraised using an Indigenous quality assessment tool and were synthesised via inductive thematic analysis informed by an ecological framework.

**Results:**

The search identified 968 studies with 7 meeting the inclusion criteria. Key factors influencing breastfeeding and infant feeding practices of Aboriginal women included cultural practices, normalisation of bottle feeding, shame associated with breastfeeding in public, access to culturally safe nutrition education, support services and health professionals, family/partner support, knowledge of the benefits of breastfeeding, experiences with previous babies and concern that the baby was not getting enough milk.

**Conclusion:**

The perspectives of Aboriginal and Torres Strait Islander women must be considered when providing breastfeeding and infant feeding advice. This can be achieved through Aboriginal and Torres Strait Islander people designing, implementing, and leading the delivery of education and information regarding breastfeeding and health infant feeding practices that have been influenced by the priorities of Aboriginal and Torres Strait Islander communities.

## Introduction

Nutrition during first 1000 days of life is critical for an infant’s growth, development and lifelong health. Breastfeeding is the optimal method for feeding an infant, as it is nutritionally complete, clean, free of charge and contains important digestive and immunological factors [1]. There are numerous studies demonstrating the benefits of breastfeeding for both babies and mothers, with breastfeeding duration associated with reduced morbidity and mortality [1, 2]. Breastfed infants have a lower risk of potentially life-threatening diarrhoeal and respiratory infections, otitis media, sudden infant death syndrome and childhood leukemia [3–6]. Breastfeeding contributes to an infant’s immunity, cognitive functioning and protects against obesity and type 2 diabetes later in life [7, 8]. Likewise, for women, breastfeeding assists with weight reduction after pregnancy [1, 9, 10] protects against breast, uterine and ovarian cancer, and reduces the risk of developing rheumatoid arthritis, coronary heart disease and type 2 diabetes [1, 9, 11]. The Australian Infant Feeding Guidelines recommend exclusive breastfeeding for around six months, after which time appropriate solid foods can be introduced while breastfeeding continues until the infant is twelve months of age, or longer if desired [12]. These guidelines align with those of the World Health Organization (WHO), who recommend continued breastfeeding for up to two years and beyond [13].

Despite strong support for breastfeeding in national and international policies and guidelines [12, 13], many infants are not optimally breastfed. Globally, 60% of babies do not receive breastmilk within the first hour of life, and only 41% of infants aged 0–6 months are exclusively breastfeed [13]. In Australia, it has been reported that 93% of infants receive some breastmilk but only 29% are exclusively breastfed to 6 months of age [14]. Within Australia, there are population subgroups with lower breastfeeding rates than the general population [15]. The 2018–2019 National Aboriginal and Torres Strait Islander Health Survey found that although the majority (87%) of Aboriginal and Torres Strait Islander children aged 0–2 years had received some breastmilk only 12% had been breastfed for 6–12 months and only 7% for 12 months or more [16]. The findings of a recent systematic review suggest that, with the exception of women living in remote areas, rates of breastfeeding initiation, maintenance and exclusivity are consistently lower among Aboriginal and Torres Strait Islander women compared with non-Indigenous mothers [17].

The number of Aboriginal and Torres Strait Islander babies born in Australia increased between 2009 and 2019, representing the future of the world’s oldest continuous culture [18]. However, health inequities persist, with child mortality rate twice that of non-Indigenous Australians and life expectancy 8 years lower than for other Australians [19]. These persistent, unacceptable health disparities are underpinned by historical and ongoing processes of colonisation, dispossession, transgenerational trauma, racism and socioeconomic disadvantage [20].

Before Australia was colonised, Aboriginal and Torres Strait Islander peoples lived healthy, sustainable and socially connected lives. Breastfeeding was a routine part of pre-colonial life, with some reports of babies being breastfed for up to 4 years [21]. Colonisation significantly disrupted traditional child rearing practices and the transmission of cultural knowledge [22], likely contributing to the lower breastfeeding rates among Aboriginal and Torres Strait Islander women today, particularly in urbanised areas.

The Australian government has introduced numerous policies and strategies aiming to ‘Close the Gap’ in health outcomes between Aboriginal and Torres Strait Islander peoples and non-Indigenous Australians [23, 24]. Within this policy agenda, maternal and infant health is a key priority. The National Aboriginal and Torres Strait Islander Health Plan recommends the implementation of strategies to increase initiation and duration of breastfeeding along with targeted nutrition programs for mothers and infants in the early stages of an infant’s life [23]. Furthermore, the Australian National Breastfeeding Strategy: 2019 and Beyond, includes Aboriginal and Torres Strait Islander mothers and babies as a priority population and commits to a strength-based approach that is collaborative with Aboriginal organisations and communities [25]. Despite these policy recommendations, significant improvements in breastfeeding initiation and maintenance among Aboriginal and Torres Strait Islander women have yet to be realised.

Aboriginal and Torres Strait Islander peoples’ concept of health is vastly different to that of the widely accepted western medical model. Aboriginal and Torres Strait Islander health is viewed holistically and encompasses more than the functionality of the physical body, with the external environment and connections to land and culture equally important [26]. Any policy or strategy to promote breastfeeding among Aboriginal and Torres Strait Islander families must be developed with the views, values and voices of Aboriginal and Torres Strait Islander people at its centre.

To design effective policy to increase breastfeeding rates, it is important to understand women’s perspectives about breastfeeding and the factors that influence their infant feeding decisions. A systematic review of international evidence on the factors influencing infant feeding choices among women from nine different countries identified that women’s own views, advice from family, friends and health professionals, sociocultural norms, and media representations were all key determinants of breastfeeding [27]. Another international review suggested feelings of guilt, shame and frustration resulted in mothers switching to formula feeding and avoiding ongoing postnatal care [28]. It is unclear, however, whether these findings also apply to Aboriginal and Torres Strait Islander women as Aboriginal knowledge, worldviews and lived experiences are very distinctive and need to be acknowledged as being a vital part of what it means to be a First Nations person.

To our knowledge, relevant qualitative research documenting Aboriginal and Torres Strait Islander perspectives and experiences on the factors that may enable or constrain breastfeeding has not previously been synthesised. Therefore, this review aims to answer the following question: What are the factors influencing breastfeeding and infant feeding decisions for Aboriginal and Torres Strait Islander parents and carers? A secondary aim of this review was to critically appraise the available evidence from an Indigenous perspective.

## Method

In order to provide evidence for the Australian context, a systematic review of the peer-reviewed qualitative literature was undertaken to synthesise the available evidence on the factors influencing infant feeding for Aboriginal and Torres Strait Islander women. The review followed the Preferred Reporting Items for Systematic Reviews and Meta-Analyses (PRISMA) guidelines to ensure transparency of methods and reporting [29, 30] and the protocol was registered with PROSPERO (CRD42021230949). The review was led by an Aboriginal researcher (FM) who combined conventional systematic review methodology with Indigenous research methods which privilege Aboriginal and Torres Strait Islander voices and ways of knowing, being and doing. We applied an Indigenous lens to the critical appraisal of included studies through team yarning. Yarning is an established informal Indigenous research method involving both structured and unstructured dialogue carried out over several sessions [31]. The review team included two Aboriginal researchers (FM and TW), a Torres Strait Islander researcher (KH) and a non-Indigenous researcher with extensive experience working in the Aboriginal community-controlled health sector (JB).

### Search strategy

Four academic databases: MEDLINE, CINAHL (via EBSCOHost), and the Health and Indigenous Collections within Informit were searched for peer-reviewed literature. Google Scholar and the Australian Indigenous Health Infonet were searched to identify additional studies. The Sample, Phenomenon of Interest, Design, Evaluation, Research Type (SPIDER) framework was used to create a list of search terms (Tables 1 and 2) for the Medline search strategy.

**Table 1:** Database Search Terms

Sample	Phenomena of Interest	Design	Evaluation	Research Type
Aborigin*	Wean* Matern*	Yarn* Interview*	Perspective* Enable*	Qualitative
“Torres Strait Island*”	Pregnan* Breastfeed*	“focus group*” “story telling”	Barrier* Experience*	Indigenist
Indigen*	“bottle feed*” “infant formula”	* Indicates truncation	Determine* Influence*	“mixed methods”
“First Nation*”	“first food*” “solid food*” “infant feed*” “infant food*” “infant drink*” “child rearing” “child raising” “raising children”		Facilitat* Support* Factor*	Narrative*

*Indicates truncation

**Table 2:** MEDLINE search strategy

1	TI OR AB	Aborigin* OR “Torres Strait Island*” OR Indigen* OR “First Nation”
2	MH	Oceanic Ancestry Group+
3	1 OR 2	
4	TI OR AB	wean* OR matern* OR pregnan* OR breastfeed* OR “bottle feed” OR “infant formula” OR “first food*” OR “solid food*” OR “infant feed” OR “infant food*” OR “infant drink*” OR “child rearing” OR “child raising” OR “raising children”
5	MH	Breast Feeding + Bottle Feeding+
6	4 OR 5	
7	TI OR AB	Yarn* OR Interview* OR “focus group*” OR storytelling OR narrative OR qualitative OR Indigenist “mixed methods” indigenist OR “mixed methods”
8	MH	Focus Group + Qualitative Research+
9	7 OR 8	
10	TI OR AB	Perspective* OR enable* OR barrier OR experience* OR determin* OR influence* OR facilitator* OR support* OR factor*
11	MH	Causality + OR Sociological Factors+
12	10 OR 11	
13	3 OR 6 OR 9 OR 12	

*Indicates truncation

Where possible, relevant subject headings were included in each database search. Each set of search terms and subject headings were combined with the Boolean operator ‘OR’, then results of each set were combined with the operator ‘AND’. Database searches were undertaken in January 2021 and no date limits were applied.

### Inclusion criteria

Studies were considered eligible for inclusion in the review if they (1) included Aboriginal and/or Torres Strait Islander women and their families (2) were about infant feeding, including breastfeeding, formula feeding or introduction of solid foods, (3) incorporated the perspectives, enablers, barriers and factors influencing breastfeeding and/or infant feeding decisions, and (4) used a qualitative research approach and/or Indigenous research methodologies such as yarning, storytelling, focus groups and individual discussions.

### Study screening and selection

Studies identified through the above search strategy were uploaded into Covidence systematic review software, which was used separately by two researchers (FM and JB) to screen the titles and abstracts for relevance. The full text of eligible studies was then independently reviewed by the same two researchers against the inclusion criteria. Articles not meeting these criteria were excluded. The results were then discussed with the final seven studies agreed on for inclusion in the review.

### Quality assessment

Quality appraisal of the included articles was undertaken by two Aboriginal researchers (FM and TW). As this study aimed to critique the available evidence from an Indigenous perspective, we elected not to use a general qualitative research critical appraisal tool and instead applied the Centre of Research Excellence in Aboriginal Chronic Disease Knowledge Translation and Exchange (CREATE) Aboriginal and Torres Strait Islander Quality Appraisal Tool [32, 33]. The quality appraisal tool consists of fourteen questions that assess research conducted with Aboriginal and Torres Strait Islander people. The purpose of the CREATE quality appraisal tool is to ensure that such research is conducted in a culturally appropriate way that is respectful of values and beliefs and enables self-determination. Included in the CREATE tool is an assessment of the degree to which the studies were guided by an Indigenous research paradigm. This is important as integration of Indigenous knowledge into research actively echoes Indigenous voices as a direct reflection of the community and the surrounding environment [34]. Studies were considered high quality if at least 9 of the 14 appraisal questions were endorsed, moderate quality if 5–8 of the questions were satisfied, and low quality if authors provided evidence in the text for less than 5 of the appraisal questions.

### Data extraction and analysis

A data extraction spreadsheet was developed in Microsoft Excel to record the key characteristics of included studies. These included the study setting, study design, number of participants and participants demographics, the authors’ key thematic findings regarding factors influencing infant feeding and the recommendations for policy and practice as made by the authors. Details were also extracted regarding the level of Indigenous involvement in the research. Data extraction and quality assessment were undertaken independently by two Aboriginal authors (FM and TW) and consensus was reached through two yarning sessions.

**Fig. 1:**
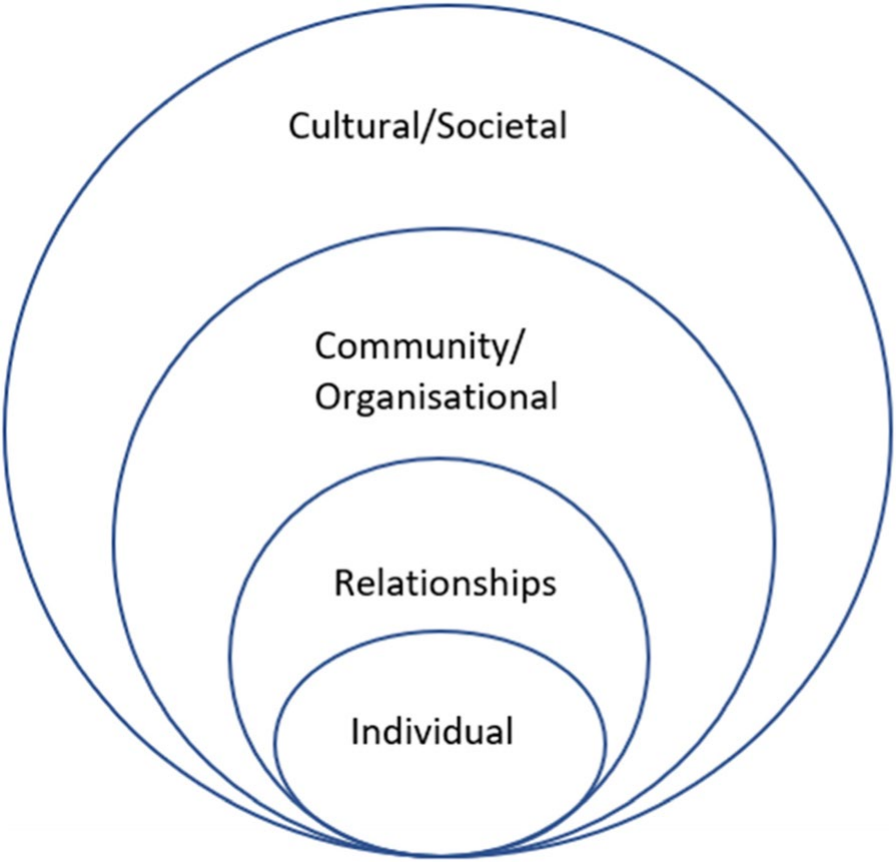
Adapted ecological framework [36]

The results of included studies were analysed using an integrated qualitative thematic analysis approach. Studies were uploaded into NVIVO qualitative analysis software [35]. Line by line inductive coding of the results section of each study, including participant quotes, was undertaken independently by two researchers (FM and JB). Codes that were similar were grouped together to develop themes, with agreement on a final set of themes and subthemes. The themes were arranged according to an ecological framework [36] which acknowledges that an individual does not exist in a silo but is part of a broader community and society where external factors influence their behaviour and overall wellbeing (Fig. 1).

As such, the socioecological framework aligns with the Aboriginal and Torres Strait Islanders holistic approach to health and wellbeing.

## Results

**Fig. 2:**
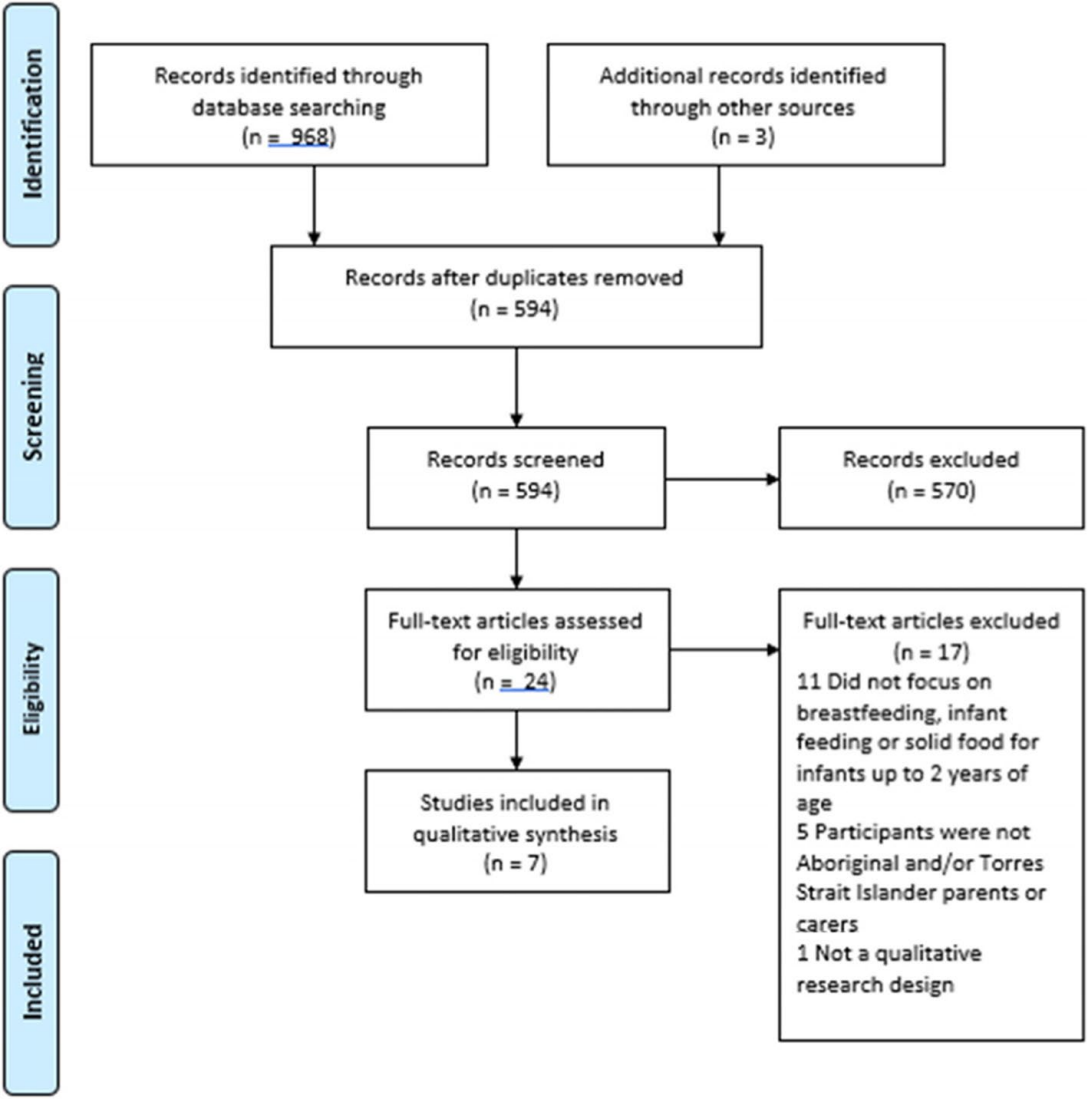
PRISMA Flow diagram of included and excluded studies

The database searches identified 594 studies after duplicates were removed. There were twenty-four full-text articles that were assessed for eligibility, of which 7 journal articles were considered eligible for inclusion in this review based on the selection criteria (Fig. 2).

The included studies were published between 1997 and 2016, with the majority (*n* = 5/7) published between 2012 and 2016. We found two studies from Victoria, two from Queensland, one from Western Australia, one from New South Wales and one from the Northern Territory. Four studies were undertaken in an urban area, two within a rural area and one in a remote area. The studies used various qualitative designs and methods for data collection including yarning sessions, interviews, and focus groups. There were 405 Aboriginal and/or Torres Strait Islander People who participated in the studies. Most studies (*n* = 4/7) involved female participants aged 16–36 years and focused on the perspectives of women, three also included male participants. Table 3 provides an overview of the characteristics of included studies.

**Table 3:** Characteristics of included studies

Author, year	Location (Rurality)	No. & type of participants (gender)	Indigenous Participants %	Age Groups (years)	Key Findings
Eades et al.,2000 [37]	Urban Western Australia	274 mothers (female)	100%	Not provided	Factors supporting breastfeeding: knowing it is best for baby, cheaper, convenient; previous breastfeeding experiences; and advice from health professionals. Factors constraining breastfeeding: fear baby is not getting enough milk; breast problems, mother stressed/sick/tired/shamed; mothers’ preference; and maternal smoking.
Foley et al., 2013 [38]	Urban Queensland	21 parents (20 female; 1 male)	100% Female Unsure of male	Not provided	Factors supporting breastfeeding: infant feeding strategies developed to fit within social circumstances; enjoying closeness with baby, support in hospital. Factors constraining breastfeeding: pain, frustration, difficult birth, feeding problems, concern about milk supply, nurse offering formula; and lack of breastfeeding support in hospital.
Foley et al., 2016 [39]	Urban Queensland	20 mothers (female)	100%	16–39	Factors supporting breastfeeding: empathetic, trusting relationships with health professionals, continuity of care, proactive health sector support, home visits/phone calls, tailoring advice to mother’/infants’ specific needs. Factors constraining breastfeeding: lack of breastfeeding support in hospital, care where no relationship established.
Helps et al., 2015 [40]	Rural New South Wales	15: 8 first time mothers, 5 Aboriginal health workers, 2 community breastfeeding champions (female)	100%	18–26	Factors supporting breastfeeding: knowing breastfeeding is the best thing for baby, cheaper, easier for night feeding and promotes weight loss. Family and community support for breastfeeding. Factors constraining breastfeeding: lack of knowledge about breastfeeding, normalisation of formula feeding, marketing of toddler formula, overcrowded housing, shame about breastfeeding in public.
Holmes et al., 1997 [41]	Urban Victoria	Approx. 35 Parents, pregnant women, men (male and female)	Not clearly stated	17–52	Factors supporting breastfeeding: belief that it is a natural, accepted and a traditional practice, cost. Factors constraining breastfeeding: embarrassment about feeding in public, normalisation of formula perception that breastfeeding is inconvenient; belief that formula is as good as breastmilk; sore nipples; worries about milk supply; jealous partners.
Kruske et al., 2012 [42]	Remote Northern Territory	15 mothers (female)	100%	15–29	Breastfeeding commonplace and babies rarely offered solid foods before 8 or 9 months of age. Factors influencing infant feeding: traditional childrearing practices, kinship relationships, cultural practices and beliefs.
Myers et al., 2014 [43]	Rural/Urban Victoria	35 parents (13 female; 22 male) 45 early childhood practitioners (predominantly female)	75%	Not provided	Factors supporting breastfeeding: knowing it is best for baby, cheaper, enjoy bonding with baby, support from Aboriginal health service/ Aboriginal health workers. Factors constraining breastfeeding: reliance on sweet drinks and bottles, unsupportive/jealous partners; shame/embarrassment.

According to the Aboriginal and Torres Strait Islander Quality Appraisal Tool, two studies were rated as high quality, four studies were considered moderate quality and one study was considered low quality (Table 4).

**Table 4:** Results of Aboriginal and Torres Strait Islander quality appraisal tool [29, 30]

	Indigenous governance				Respect for cultural and intellectual property				Capacity building			Beneficial outcomes			Overall assessment
	Q1	Q2	Q3	Q4	Q5	Q6	Q7	Q8	Q9	Q10	Q11	Q12	Q13	Q14	
Eades et al., 2000 [37]	P	Y	Y	Y	Y	N	N	Y	U	P	P	Y	U	U	Moderate
Foley et al., 2013 [38]	Y	Y	U	U	Y	U	U	U	P	Y	P	Y	N	U	Moderate
Foley et al., 2016 [39]	Y	Y	U	U	Y	U	U	U	U	Y	P	Y	N	U	Moderate
Helps et al. 2015 [40]	Y	Y	N	Y	Y	P	P	Y	Y	Y	P	Y	Y	U	High
Holmes et al., 1997 [41]	Y	Y	Y	Y	Y	U	U	U	N	Y	Y	Y	Y	U	High
Kruske et al., 2012 [42]	N	U	P	P	Y	N	N	U	U	Y	N	U	U	U	Low
Myers et al., 2014 [43]	P	Y	Y	Y	Y	N	N	U	U	P	N	P	Y	P	Moderate

Key: Y = Yes N = No P = Partially U = Unclear

Community consultation and respect for protocols, governance and research leadership was evident to some degree in most of the studies with some studies also showing evidence of capacity building. The seven included studies documented little evidence of agreements to protect Indigenous cultural and intellectual property and of the research providing beneficial outcomes for Aboriginal and Torres Strait Islander participants or communities.

### Factors influencing breastfeeding and infant feeding practices

We identified ten themes about the factors influencing Aboriginal and Torres Strait Islander women and their families breastfeeding and infant feeding practices. The themes align with the levels in the ecological framework [36], are summarised in Table 5 and are described in detail below.

**Table 5:** Summary of key themes identified and selected quotes from included studies

Ecological Level	Themes	Parents’ quotes
Cultural/Societal	Breastfeeding is an accepted cultural practice	“Well I just thought you just put the baby on your breast when it was born, that was it. I never thought about bottles.“ (37)
	Formula and bottle feeding have become normalised	“Especially with the bottle, cordial in the bottle, that’s rotting teeth, my kids got em” (38)
	Breastfeeding in public is shameful	“I’ll get too shamed out… I’ll probably just put milk in a bottle.“ (39)
Community/ Organisational	Access to culturally appropriate nutrition education	“Trained (Aboriginal) nutrition workers. That’d be awesome…really good.“ (38)
	Culturally safe and supportive maternal/infant health services and professionals	“I think a lot of the ACCHO’s really needs to be looking at having our own maternal and child health nurses ‘cos there’s just so much conflict with mainstream ones.“ (38)
Relational	Family support	“I really only talk to my family about it… cause they’re the only ones that I would listen to” (39)
	Fathers’ support during breastfeeding	“For me, personally I’m not about to see my woman hanging out her tit” (37)
Individual	Breastfeeding is cheap, clean and best for baby	“It’s just much easier and cheaper to breastfeed. I don’t want to spend money on formula.“ (40)
	Mothers’ experiences with previous babies	“[I had] bad experience with first baby – cracked nipples” (41)
	Concern that baby was not getting enough milk	“She wasn’t feeding properly, and I wasn’t getting enough milk” (40)

Quotations from participants are included to highlight the lived experiences of Aboriginal and Torres Strait Islander women and their families.

### Cultural/societal level factors

#### Theme 1: Breastfeeding is an accepted cultural practice

Aboriginal and Torres Strait Islander participants described breastfeeding as a traditional practice that should be continued; however, the influence of colonisation and the growing preference for bottle feeding was evident among some Aboriginal women. The earliest study [41] included in this review, whose participants were from an urban Aboriginal Community in Melbourne, noted that acceptance of breastfeeding was so high in the community that there was a strong belief among both men and women “that Aboriginal women ought to breastfeed” [41]. Breastfeeding was considered a common, natural practice and was something that did not warrant questioning. This is highlighted by a mother:

“” Well, I just thought you just put the baby on your breast when it was born, that was it. I never thought about bottles.”” [41].

#### Theme 2: Formula and bottle feeding have become normalised

While breastfeeding was reported to be continued for long durations among Aboriginal and Torres Strait Islander women in remote areas, some participants from studies in urban and rural areas indicated that bottle feeding had become normalised, sometimes with sugar-sweetened beverages given to babies in conjunction with formula [37, 43]. For many women in these communities, infant formula was viewed as a convenient option and a good alternative when breastfeeding was not possible. Participants spoke positively about the many formula brands to choose from, describing them as being “best for baby” [40] and a way to provide baby with all the necessary nutrients. Some participants expressed concern that sugary drinks were preferred by their infants [37, 43]. Parents reported that soft drinks and cordial were always readily available and were concerned about the dental health effects of these sugary drinks being used in babies’ bottle. These concerns are highlighted below:

“”Especially with the bottle, cordial in the bottle, that’s rotting teeth, my kids got em.”” [43].

The pervasive marketing and promotion of breast milk substitutes, as well as availability of infant formula in some maternity hospitals was another factor that influenced infant feeding choices. Some mothers were influenced by the promotion of formula, demonstrating high levels of brand recognition and preferencing formula in place of breastfeeding.

“”There’s so many good formulas you can get these days. Like I watch all them ads and I’ve been taking it all in which one I might get and stuff.”” [40]

#### Theme 3: Breastfeeding in public is shameful

Another theme related to the colonisation of traditional infant feeding practices was the perception of breastfeeding in public being taboo or shameful. Mothers living in urban and rural areas, in particular, expressed shame about breastfeeding in public, whereas mothers living in remote areas did not seem concerned about the judgement of others when they breastfed their infants whenever and wherever they were hungry. This resistance to colonial attitudes to breastfeeding in public is outlined in the quote below by both Aboriginal mothers and Aunties:

“”Balanda [white people] are always worried about the right time. We eat [our babies eat] when we are hungry.”” [42].

### Community/organisational level factors

#### Theme 4: Access to culturally appropriate nutrition education

Participants overwhelmingly expressed a preference for Aboriginal Community Controlled Health Organisations (ACCHOs) for accessing culturally appropriate health information. Specifically, participants expressed a desire for more Aboriginal-specific information and support around infant health and nutrition as they reported feeling safer accessing these services through an ACCHO in comparison to mainstream maternal and child health services.

“”I think a lot of the ACCHO’s really need to be looking at having our own maternal and child health nurses ‘cos there’s just so much conflict with the mainstream ones.”” [43].

Participants reported that their preference would be to receive infant nutrition information from a trained Aboriginal health worker. Aboriginal mothers reported that they felt more comfortable speaking with Aboriginal health staff who were already familiar with the challenges Aboriginal women experience, compared to a non-Indigenous health practitioner. This finding is highlighted further in the next theme.

#### Theme 5: Culturally safe and supportive maternal and infant health services and professionals

Cultural safety was key factor determining access to maternal and infant health services for Aboriginal women. Participants from an urban study described how they experienced limited assistance from some service providers who lacked cultural understanding in relation to working with Aboriginal mothers. An experience with an insensitive health professional can deter women from seeking breastfeeding support or from accessing maternal and child health services in the future as outlined by the following participants:

“”With my first one she wanted me to buy all this expensive stuff and you couldn’t afford it…that was just unreal. I haven’t taken any of my others to a welfare centre and I keep getting these letters telling me off, but no way would I take them there…”” [41].

“”[in hospital] they never really asked [if I would breastfeed] because I said I was going to bottle feed him…so I could have a rest, but then when I got home [2 days later] I put him on the breast…because I was at home, comfortable.”” [38].

Not all interactions with the mainstream health system were reported to be negative. Participants from another urban study expressed positive experiences with maternal and child health care providers, including support provided in relation to breastfeeding and infant feeding. One participant, who received home visits to help establish breastfeeding, described how “” [Clinic nurses] would come here almost every week, I think. They would stay for ages when they came over here… They were really, really good. They helped a lot…”” [39].

### Relationship level factors

#### Theme 6: Family support

Family support was found to be a major influence on breastfeeding and infant feeding practices among Aboriginal women. Where the family was supportive it was reported that breastfeeding increased and, where families were not supportive this created a barrier to breastfeeding. This is further supported by the following quotation from one participant in an urban study who recalled how her mother intervened by introducing formula when her baby became unsettled.

“” He started getting – like he wouldn’t bring up his wind and crying; just constantly screaming…So my mum just went and got formula and made him a bottle. He drank the whole thing, done a big burp and went to sleep for like five hours.”” [38].

#### Theme 7: Fathers’ support during breastfeeding

The attitude of the infant’s father was another key factor influencing whether a woman chose to breastfeed. Many participants reported that they felt fathers were not supportive of breastfeeding, especially breastfeeding in public [40, 41, 43]. Sexualisation of breasts was reported to be a common issue, with some reporting that there was an element of jealousy experienced by fathers when mothers would engage in breastfeeding, as the women’s breasts were perceived to belong to the man and not the infant and therefore should not be shown in public [41]. It is important to note that not all men were unsupportive of their partners breastfeeding. There were also women who reported that they had supportive partners who understood the importance of breastfeeding, further highlighting that the attitudes of the father can be a key determinant of infant feeding decisions.

### Individual level factors

#### Theme 8: Breastfeeding is cheap, clean, and best for baby

Many women demonstrated knowledge of the health benefits of breastfeeding and reported these as a key factor influencing their decision to breastfeed their children. Most participants who had breastfed also mentioned the economic benefits and convenience of breastfeeding, citing “”no sterilisation of bottles”” [37] as a key benefit. Many mothers also made the overall statement that breastfeeding was “the best” option for their baby, as demonstrated by the following quotation.

“”I did it ‘cos I heard it’s the best thing for the baby.”” [43].

However, some women reported that, although they knew that breastfeeding was the best option, they found that they could not breastfeed due to various circumstances. These women reported struggling with breastfeeding even though they were provided with support and advice from health professionals.

#### Theme 9: Mothers experiences with previous babies

Participants’ past experiences with infant feeding strongly influenced their decision to breastfeed or not. For mothers who had positive past breastfeeding experiences, this encouraged them to breastfeed their newborn baby [37, 39]. Conversely, several women cited a “”bad experience with first baby”” [37] as the reason they did not breastfeed their subsequent children. Specifically, participants reported that unpleasant experiences of physical difficulties, such as cracked nipples or mastitis impacted their future breastfeeding decisions as outlined below by a mother from the community:

“” I didn’t last long though. My milk dried up and I got mastitis.”” [43].

However, issues such as mastitis did not always deter women from breastfeeding all together. Some participants within an urban area reported that they were able to successfully continue breastfeeding despite experiencing problems with establishing breastfeeding when they were provided with appropriate information and support [38]. This finding further highlights the importance of having access to culturally safe services and health professionals, as outlined in Theme 5.

#### Theme 10: Concern that baby was not getting enough milk

Among several participants, there was a perception of short milk supply which prompted mothers to choose bottle feeding [38, 41]. Some mothers, who successfully established breastfeeding, reported that they felt reassured by the fact that bottle feeding enabled them to quantify how much milk the baby was consuming. Several participants reported that even though they tried to breastfeed, they felt as though they had a “”short milk supply”” and “”baby needs more food”” [37], so switched to formula.”” For other women, expressing breastmilk into a bottle was a comforting option. For example:

“” …I was still worried that he was not getting enough milk even though it [breast] was full and it was coming out right. And so just to make myself feel good to know that he is getting what he wants, I expressed a bottle for him so that he could have a bottle like twice a week.”” [38].

## Discussion

This is the first systematic review of qualitative studies reporting the factors that influence breastfeeding and infant feeding practices for Aboriginal women. Based on the seven studies identified, which represent the voices of approximately 400 Aboriginal and Torres Strait Islander participants, ten themes were identified across the individual, relational, community and cultural/societal levels of the ecological framework. At the individual level, the knowledge that breastfeeding is cheap, clean, and best for baby; mothers’ experiences with previous babies; and health concerns were key factors influencing infant feeding decisions. At the relational level, family and partners were a key influence, while health practitioners and health services were important at the community level. Finally, at the cultural/societal level, the fact that breastfeeding is an accepted traditional practice in some communities was a key enabler, while in others, the fact that infant formula and bottle feeding have become normalised and breastfeeding in public was considered shameful were found to be key barriers. The key findings regarding the cultural/societal, community/organisational, relational, and individual factors influencing breastfeeding are discussed below.

### Cultural/societal factors

A key finding of this review was that, for many women in remote communities, breastfeeding remains an accepted traditional practice. This finding is supported by quantitative evidence which suggests that, in remote communities, breastfeeding rates among Aboriginal women are higher than among non-Aboriginal women [44]. An Indigenous midwife practicing in the remote Kimberley region of Australia [45], noted that Aboriginal women learn about breastfeeding through observation and not by asking questions as this is considered rude. Traditional practices such as the smoking ceremony have also been shared with non-Indigenous mothers experiencing difficulty with lactation who have their breasts ‘smoked’ to encourage the supply of breastmilk [45]. Findings of previous studies have also outlined the acceptance of breastfeeding among Aboriginal women and identified that it contributes to their perception of being a mother [46, 47]. Aboriginal women have a strong connection to culture and often report breastfeeding to be an influential part of their lives, flowing into their expression of maternity [45]. Just as the ‘birthing on Country’ movement has demonstrated improved outcomes for Aboriginal mothers and babies [48], a focus on connection to culture offers a unique opportunity for strengths-based approaches to increase breastfeeding among Aboriginal women.

The findings from this review demonstrate that, while many Aboriginal women assume breastfeeding to be a culturally acceptable practice, the ongoing impact of colonisation is experienced by some Aboriginal women through feeling shameful about breastfeeding in public. The colonisation of traditional Indigenous food systems has been well documented [49], however it is often recognised that this colonisation extends to breastfeeding. Experiences of shame or awkwardness in public are shared with non-Aboriginal women, who have reported feelings of moral judgement if breastfeeding in public [28]. Taken together, these findings suggests that in order to improve breastfeeding, interventions must go beyond targeting women alone and must extend to broader society.

The infant formula industry is big business and controlled by powerful companies who have a financial incentive to undermine breastfeeding [50]. For this reason, the International Code of Marketing of Breastmilk Substitutes was adopted by the World Health Assembly in 1981 [51]. Ongoing implementation and monitoring of the code is required to ensure the protection and promotion of breastfeeding, especially in urban and rural Aboriginal communities where formula feeding is becoming normalised. The impact of colonisation, including by the infant formula industry, have presented Aboriginal mothers with some cognitive challenges regarding decisions about their infants’ nutrition. This has led to early cessation of breastfeeding, which has implications for long-term health, resulting in a feedback loop of poor health that is accentuated by ongoing colonial practices [45, 52]. The more society normalises breastfeeding as an accepted practice, the healthier Aboriginal and Torres Strait Islander infants will be during their development and across the life course. Monitoring the activities of the breastmilk substitute industry with regard to Indigenous women is an important area for future research.

### Community/organisational factors

A key finding of this review is that Aboriginal mothers have expressed a preference for attending ACCHOs and to be guided by Aboriginal health workers who have specialised knowledge about infant feeding that is both relevant and culturally sound. A preference for receiving maternal and child health care from ACCHOs is supported by the findings of a recent review of quantitative studies which found that attending an Aboriginal specific service was associated with increased breastfeeding initiation and maintenance [17]. ACCHOs provide holistic, culturally appropriate primary health care, health education and information, for Aboriginal and Torres Strait Islander people [53]. The findings of this review suggest that trained Aboriginal health staff within ACCHOs, with specific expertise in infant nutrition, would enable Aboriginal mothers to access maternal and child health services [43]. Supporting ACCHOs and Aboriginal health staff to provide culturally appropriate breastfeeding support is an important strategy that should be a priority for Closing the Gap policy.

Education and support around infant feeding that is specifically for Aboriginal mothers, delivered through culturally appropriate programs led by trained Aboriginal staff, who can build trusting relationships with mothers, is a logical extension to best practice approaches to pregnancy care for Aboriginal women [54]. Regular postpartum contact with Aboriginal mothers through sustained home visiting, have resulted in high levels of satisfaction among Aboriginal mothers with reports of positive impacts on breastfeeding duration which are now being empirically tested [55]. The findings in this review regarding the importance of targeted, culturally safe services and programs for Aboriginal mothers is shared with Maori mothers who have also experienced limited availability of culturally appropriate maternity information and support while being reluctant to attend mainstream hospital-based maternal programs [56]. This deficit further emphasises the need for tailored breastfeeding support and information for Aboriginal families to address access and cultural safety issues while encouraging autonomy.

### Relational factors

Family support is a key determinant of breastfeeding. Our findings suggest that positive, supportive family environments enabled breastfeeding practices and breastfeeding decreased when family support was missing. Within Aboriginal families, breastfeeding is not only a source of nourishment but also security, protection and a connection to kin and culture thereby, grounding the infant in place from their early stages in life [57]. The support provided by family members, according to the findings of this review, ranged from nutrition advice while the mother was pregnant through to the overall care of the infant after birth [54]. Further exploration is needed regarding culturally sensitive approaches to support breastfeeding for Aboriginal women with little family support or for those who have grown up in families in which formula feeding has become normalised.

This review, along with previous reviews [27, 58, 59] found that fathers have a crucial influence on women’s infant feeding decisions and that, having a supportive partner helped to enable breastfeeding. There is evidence to suggest that being partnered is associated with higher breastfeeding initiation among Aboriginal women [17]; however, this review found that not all fathers were supportive of their partners breastfeeding as the breast was perceived, in some instances, as a sexual body part belonging to the partner and some fathers did not want their partner to breastfeed in public where other men could see them [43].

In Aboriginal culture, the traditional distribution of roles into men’s and women’s business allowed for the understanding of place within the Community along with the kinship system of families [60]. The role of Aboriginal fathers extends beyond their immediate children as the men have a responsibility to their extended family and Community to be respected, strong role models [60]. Having a safe space for Aboriginal men to come together to discuss their concerns and connect with other Aboriginal men is essential for their wellbeing, and for dispelling misinformation, promoting positive role models, and connection to Community [61].

The findings of this review align with previous studies in relation to the challenges facing Aboriginal men trying to find a place as a father and role model in community post-colonial society [61]. Traditionally, knowledge regarding the role of men in child-rearing would have been passed down through the generations. Colonisation disrupted traditional knowledge systems and, therefore, men’s groups have become the contemporary method for Aboriginal men to come together to exchange knowledge [60]. There is evidence to suggest that providing breastfeeding education directed at fathers can improve breastfeeding outcomes for mothers [59]. This is a promising approach which should be considered alongside breastfeeding education and support for Aboriginal women.

### Individual

The individual-level factors, identified in this review, that encouraged breastfeeding included the knowledge that it was the best option for the baby and was more affordable than bottle feeding. Qualitative research with Maori women had also highlighted that the fact that breastfeeding is cheap and no preparation needed, especially during the night, make it an attractive option for mothers [56]. Even though breastfeeding is often viewed as cheaper and convenient, not all mothers choose to breastfeed their infants. A study by Brown et al. [62], found that Aboriginal women in South Australia had high rates of breastfeeding after being discharged from hospital however, at 26 weeks postpartum the number significantly decreased. The main reported reasons for switching to formula were perceptions of insufficient milk supply and physical concerns such as sore nipples and mastitis, which mirror the findings of this review. A key learning from this review is that individual breastfeeding education and support for Aboriginal women must be considered and addressed alongside the relational, community, cultural and broader societal determinants of breastfeeding.

### Strengths and limitations

A key strength of this review was that it was Aboriginal led. This is important because it changes the dominant narrative of Indigenous research being undertaken by non-Indigenous researchers. The use of the Aboriginal and Torres Strait Islander Quality Appraisal tool is a further strength of the current review as it provides guidance as to whether cultural safety and data sovereignty protocols were considered throughout the research process. A further strength of our research approach is the way in which Western and Indigenous research methods were combined. In undertaking this review, our predominantly Indigenous review team harmonised the Western systematic review process and Indigenous ways of knowing and doing through the incorporation of Indigenous research methods which are a more fluid approach. Indigenous research methodologies can be easily dismissed as they ultimately do not fit with the Western scientific approaches, but this does not mean that they are less valid [63]. While our search strategy followed a recommended Western structure [29], Indigenous research methods, such as yarning, were incorporated into the study selection and quality appraisal process.

This review also has several limitations. Despite a thorough search strategy, there were only seven studies identified, the most recent of which was published in 2016, indicating that this is an under-explored topic. Another important limitation is that not all Australian states and territories were represented and there were no studies based in the Torres Strait Islands. Most of the studies had small sample sizes, which is common in qualitative research, thus we caution readers that the perspectives reported in this review may not represent the diverse views and experiences of all Aboriginal and Torres Strait Islander peoples. Even though the studies used widely accepted qualitative research methods, greater inclusion of Indigenous research methodologies, especially as the research focused on First Nations peoples, would have strengthened the methodological and cultural rigor [64]. Furthermore, most of the studies were led by non-Indigenous researchers, with Aboriginal co-investigators included in some instances. Only two studies were considered high quality according the Aboriginal and Torres Strait Islander quality appraisal tool; however, it is important to acknowledge that this tool was published in 2020 and all included studies were completed before its’ development. No themes were generated based on the findings of low-quality studies alone.

Despite these limitations, drawing together the available studies in this review enabled a broader perspective on the topic of infant feeding and included the voices of over 400 Aboriginal and Torres Strait Islander participants.

We suggest that the CREATE quality appraisal tool should be incorporated in all systematic reviews focusing on Aboriginal and Torres Strait Islander people and that the CONSIDER statement guide the reporting of research involving Indigenous people, both in Australia and internationally [65]. The position that all researchers, both Indigenous and non-Indigenous, should take when working with Aboriginal and Torres Strait Islander communities is to ensure that the Indigenous voice is heard and respected and that all communication and engagement is undertaken in a reciprocal and respectful manner.

## Conclusion

The findings of this review suggest that cultural, societal, community, relational and individual factors influence breastfeeding and infant feeding practices of Aboriginal and Torres Strait Islander women and their families. These factors are interrelated and all need to be considered when designing policy and programs to support Aboriginal and Torres Strait Islander families. For breastfeeding to remain a culturally accepted practice, policy and programs need to be more considerate of the worldviews of our First Nations people. This can be achieved through community-designed and led programs that incorporate Aboriginal ways of doing and culturally appropriate health promotion activities that the community can develop and implement. The support of both the partner and the family contribute to a longer duration of breastfeeding along with culturally appropriate information and education, provided by local ACCHOs and trained Aboriginal health staff. This is viable through the tangible actions of increased funding that supports community-led initiatives along with the training of Aboriginal maternal health workers and midwives. Future research for breastfeeding and infant feeding among Aboriginal and Torres Strait Islander women needs a greater use of Indigenous research methodologies, where research is based on Aboriginal ways of knowing, being and doing and are inclusive of local community priorities. Such research can help identify community-led initiatives for the best approaches to improve the acceptance of breastfeeding practice for Aboriginal and Torres Strait Islander mothers and their families.

## Data Availability

All included articles are publicly available. All data generated or analyzed during this study are included in this published article.
